# Prevalence and factors correlating with hyperoxia exposure following cardiac arrest – an observational single centre study

**DOI:** 10.1186/1757-7241-21-35

**Published:** 2013-05-02

**Authors:** Annika Nelskylä, Michael J Parr, Markus B Skrifvars

**Affiliations:** 1 Department of Anaesthesiology and Intensive Care, Helsinki University Central Hospital, University of Helsinki, Topeliuksenkatu 5, PL 266, Helsinki, Finland; 2 Intensive Care Unit, Liverpool Hospital, Liverpool Hospital, Elizabeth street, Liverpool, NSW 2170, Australia; 3 University of New South Wales, Sydney, Australia

**Keywords:** Cardiac arrest, Hyperoxia, Mechanical ventilation

## Abstract

**Purpose of the study:**

Arterial hyperoxia during care in the intensive care unit (ICU) has been found to correlate with mortality after cardiac arrest (CA). We examined the prevalence of hyperoxia following CA including pre-ICU values and studied differences between those exposed and those not exposed to define predictors of exposure.

**Materials and methods:**

A retrospective analysis of a prospectively collected cohort of cardiac arrest patients treated in an Australian tertiary hospital between August 2008 and July 2010. Arterial blood oxygen values and used fractions of oxygen were recorded during the first 24 hours after the arrest. Hyperoxia was defined as any arterial oxygen value greater than 300 mmHg. Chi-square test was used to compare categorical data and Mann–Whitney *U*-test to continuous data. Statistical methods were used to identify predictors of hyperoxia exposure.

**Results:**

Of 122 patients treated in the ICU following cardiac arrest 119 had one or several arterial blood gases taken and were included in the study. Of these, 49 (41.2%) were exposed to hyperoxia and 70 (58.8%) were not during the first 24 hours after the CA. Those exposed had longer delays to return of spontaneous circulation (26 minutes vs. 10 minutes) and a longer interval to ICU admission after the arrest (4 hours compared to 1 hour). Location of the arrest was an independent predictor of exposure to hyperoxia (P-value = 0,008) with out-of-hospital cardiac arrest patients being more likely to have been exposed (65%), than those with an in-hospital (21%) or ICU (30%) cardiac arrest. Out-of-hospital cardiac arrest patients had higher oxygen concentrations to the fraction of inspired oxygen ratios.

**Conclusions:**

Hyperoxia exposure was more common than previously reported and occurred more frequently in association with out-of-hospital cardiac arrest, longer times to ROSC and delays to ICU admission.

## Introduction

The mortality following a sudden cardiac arrest (CA) is high [1,2]. Following return of spontaneous circulation a pathophysiological state named post cardiac arrest syndrome ensues and treatment focuses on stabilising circulation and alleviating the evolving neurological injury [3]. Factors associated with worsening neurological injury after cardiac arrest includes hyperthermia, hyperglycaemia, and hypercapnia [4-6]. Recently hyperoxia exposure after cardiac arrest has also been suggested to be associated with poor outcome [7]. Hyperoxia may increase free radical production, triggering cellular injury and apoptosis [3]. Another study by Bellomo and colleagues did not show any correlation between arterial hyperoxia and outcome following cardiac arrest [8]. Both these studies did not include important resuscitation data, such as initial rhythm and time to return of spontaneous circulation (ROSC) that may have influenced outcome [3]. In addition both studies included only oxygen values measured in the intensive care unit (ICU), and this might underestimate the true incidence of hyperoxia exposure following cardiac arrest.

The purpose of this study was to examine the prevalence of hyperoxia in an unselected sample of patients treated in the ICU of a tertiary hospital following either out-of-hospital (OHCA), in-hospital (IHCA) or ICU cardiac arrest (ICUCA). In this single centre trial we aimed to describe the prevalence of hyperoxia exposure and factors correlating with hyperoxia exposure immediately prior to and during the first 24 hour of intensive care.

## Material and methods

### Study setting

Liverpool hospital is a 650 bed tertiary hospital in Sydney, Australia, that serves a population of around 800,000 people. Pre-hospital care in Sydney is administered by New South Wales ambulance service and the ambulances are staffed by ambulance officers (70%) and paramedics (30%). Patients with an out-of-hospital cardiac arrest within the catchment are of Liverpool Hospital are transported to the emergency department and if found to require intensive care are admitted to the 28 bed Intensive Care Unit (ICU) of Liverpool Hospital. The ICU of Liverpool Hospital is an Intensivist run ICU with specialist availability 24/7. Cardiac arrests occurring in the hospital and in the ICU are managed by a medical emergency team (MET) described in detail elsewhere [9]. The cardiac arrest and post-resuscitation care follows international guidelines [10].

### Data collection

The collection of the data was performed between August 1^st^2008 and July 30^th^ 2010 and included all cardiac arrest patients with ROSC that survived to be admitted to the ICU. Study approval was obtained from the Human Research Ethics committee. A study involving this patient cohort has previously been published [9]. Only cardiac arrests that required chest compressions to achieve ROSC were included, excluding patients with respiratory arrests and arrhythmia requiring only defibrillation. Data was prospectively collected following the template recommended by the Utstein guidelines [11]. Cardiac arrest aetiologies were defined according to the Utstein Guidelines as myocardial ischaemia, asphyxia, trauma, lung disease, cerebrovascular disease, pulmonary embolism, cardiac failure or other. Data about the location of the arrest (OHCA, IHCA, ICUCA), and whether the arrest was witnessed or not initial rhythm, delay to return of spontaneous circulation and duration of no-flow and low-flow times were collected. Data regarding co-morbidities, patient demographics and the Apache III scores were included.

Data about respiratory management including mode of ventilation, used oxygen fraction and arterial blood gases taken before and after ICU admission during the first 72 hours after cardiac arrest were collected. Data was categorized into time points 0–1 hour and 1-24 hrs, 24-48 hrs and 48-72 hrs as recommended by the Utstein Guidelines [11]. At each time point the highest and lowest oxygen value with corresponding oxygen fractions were recorded.

### Statistical methods

For the present analysis patients were divided into two groups based on their highest arterial oxygen value measured during the first 24 hours after CA. “Hyperoxia exposure” was defined as oxygen values 300 mmHg or greater and “not exposed to hyperoxia” as arterial oxygen less than 300 mmHg. Descriptive statistics were calculated using counts and percentages for categorical variables. Median values with interquartile range (IQR) and the maximum and minimum values were calculated for continuous variables. Comparisons between patients were carried out using Chi-square test and Mann–Whitney *U* test respectively. Multivariate logistic regression was used to define independent predictors of hyperoxia exposure. Odds ratios with the corresponding 95% confidence intervals were calculated and p-values were considered significant if they were less than 0.05. All statistical analysis was performed using PASW version 18.0.

## Results

### Patient demographics

**Figure 1:**
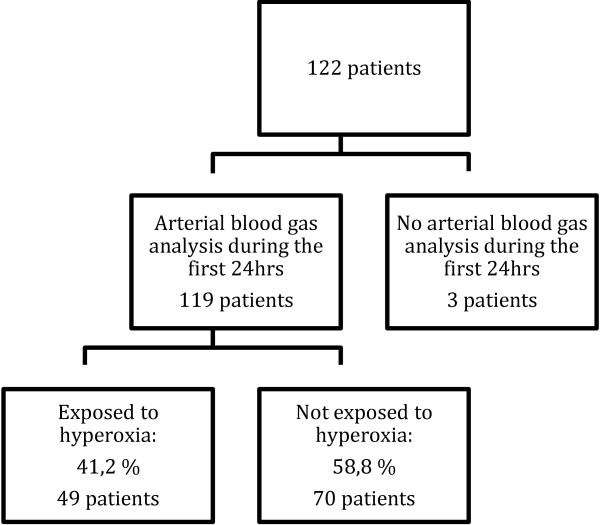
Flowchart of patient distribution.

During the study period a total of 3931 patients were admitted to Liverpool ICU, of which 122 patients were admitted following cardiac arrest. 119 of these 122 patients had one or several blood gas analysis taken during the first 24 hours after resuscitation and were included in the study (Figure 1). Mechanical ventilation was commenced in 114 patients and 5 received non-invasive ventilation. The mean age of the patients was 61 years (Table 1).

**Table 1:** Patient characteristics and factors at resuscitation in cardiac arrest patients treated in a tertiary intensive care unit based on arterial oxygen levels

	**All**	**Not exposed to hyperoxia**	**Hyperoxia exposure**	**P-value**
	**n = 119**	**n = 70**	**n = 49**	
**Arrest location**				
OHCA	43% (51)	35% (18)	65% (33)	<0,001
IHCA	40% (48)	79% (38)	21% (10)	
ICUCA	17% (20)	70% (14)	30% (6)	
**Patient characteristics**				
Age	61 (49–74)	63 (52–72)	60 (48–75)	0,633
Smoking,	24% (29)	27% (19)	20% (10)	0,400
Obesity	11% (13)	13% (9)	8% (4)	0,419
Pulmonary disease	20% (24)	17% (12)	25% (12)	0,326
Coronary artery disease	36% (43)	41% (29)	29% (14)	0,151
Diabetes	20% (24)	21% (15)	19% (9)	0,723
Pre-arrest OPC 1	68% (81)	65% (45)	75% (36)	0,604
**Resuscitation variables**				
Witnessed arrest				
No	19% (23)	17% (12)	22% (11)	0,471
Yes	81% (96)	83% (58)	78% (38)	
Assumed cause of the arrest				
Cardiac	49% (58)	49% (34)	50% (24)	0,967
Pulmonary/hypoxia	25% (29)	24% (17)	25% (12)	
Other^1^	26% (31)	27% (19)	25% (12)	
Initial rhythm				
Ventricular fibrillation/Ventricular tachycardia	40% (47)	34% (24)	47% (23)	0,205
Pulseless electrical activity	20% (24)	23% (16)	16% (8)	
Asystole	37% (44)	37% (26)	37% (18)	
Other	3% (4)	6% (4)	0%	
Median (IQR) delay to ROSC (min)	15 (5–28)	10 (5–20)	26 (12–33)	<0,001
No-flow	3 (0–10)	2 (0–5,5)	7 (0–11)	0,008
Low-flow	10 (5–20)	7,5 (3–15)	16 (7–22,5)	0,001

^1^Cause of the arrest missing for 1 patient.

### Prevalence of hyperoxia and hypoxia

Of the included patients, 49 were exposed to hyperoxia (41%) and 70 were not exposed to hyperoxia (59%) during the first 24 hours after the cardiac arrest. None of the patients receiving non-invasive ventilation were exposed to hyperoxia. Patients were commonly exposed to hyperoxia during the first hour after the arrest (n = 30) or during the following 24 hours (n = 32) (Table 2). Between 24–48 hours 83 patients had an arterial blood gas analysis taken of which none were hyperoxic and the corresponding figures for 48–72 hours were 54 and one patient. Seventeen patients (14%) were exposed to hypoxia (pO_2_-values under 60 mmHg) at any stage during the first 24 hours.

**Table 2:** **Maximal oxygen pressure (PaO**_**2**_**), inspired oxygen concentration (FiO**_**2**_**) and P/F ratio according to location of the cardiac arrest**

	**All patients**	**OHCA**	**IHCA**	**ICUCA**	**P-value**
	**n = 119**	**n = 51**	**n = 48**	**n = 20**	
**PaO**_**2 **_**max (mmHg)**					
0-1 h	180 (101–349)	265 (145–466)	124 (91–211)	177 (92–390)	0,001
1-24 h	212 (126–318)	295 (205–441)	169 (123–247)	138 (120–234)	0,001
**FiO**_**2 **_**max**					
0-1 h	100 (50–100)	100	100 (50–100)	100 (50–100)	0,001
1-24 h	100 (30–100)	100 (35–100)	100 (30–100)	90 (35–100)	0,233
**P/F -ratio**					
0-1 h	187 (105–358)	265 (145–466)	127 (93–222)	218 (136–390)	0,001
1-24 h	293 (178–425)	408 (286–518)	226 (150–333)	230 (140–389)	0,001
**Hyperoxia exposure**	41%	65% (33)	21% (10)	30% (6)	0,001
0-1 h	28% (30)	44% (20)	12% (5)	26% (5)	0,005
1-24 h	28% (32)	47% (24)	11% (5)	16% (3)	0,000

* The maximal PaO_2_ values and P/F-ratios are shown with median and IQR. The maximal Fi0_2_ values with median and maximum and minimum values.

### Patient factors and factors at resuscitation correlating with hyperoxia exposure

There were no differences between patients exposed to and those not exposed to hyperoxia concerning co-morbidities, such as lung disease, smoking habit or obesity (Table 1). Ninety-six patients had a witnessed arrest, from which 38 (78%) patients were exposed to hyperoxia and 58 (83%) were not (Table 1). There were no differences between those exposed to hyperoxia and those not exposed regarding initial rhythm or the assumed cause of the arrest (Table 1). The average delay to ROSC for the whole sample was 15 minutes and patients exposed to hyperoxia had a longer time to ROSC than those not exposed (26 min vs. 10 min, p < 0.001) (Table 1). In hyperoxic patients the no-flow (7 min) and low-flow duration (16 min) was considerably longer than in non-hyperoxic patients (2 min and 7.5 min).

### Cardiac arrest location and hyperoxia

There were significant differences between arrest location among those exposed to hyperoxia and those not exposed (p < 0.001). Of the OHCA patients 33 (65%) were more often exposed to hyperoxia when compared to IHCA patients only 10 (21%) and ICUCA patients 6 (30%) (Table 1). The delay to admission to the ICU was 4 hours for patients exposed to hyperoxia and 1 hour for those not exposed (Table 3). Median PaO_2_ was considerably higher in OHCA patients (p = 0.001) and this difference was sustained over the first 24 hours after the CA (Table 2). There was a significant difference in the highest used oxygen fraction between arrest locations during the first hour but not during the following 23 hours. The P/F-ratio (median) was similar in IHCA and ICUCA patients (226 vs. 230), and higher in OHCA patients (Table 2). When comparing OHCA and ED IHCA patients with ICUCA and other IHCA patients, OHCA or CA in ED had significantly higher oxygen values than other patients (211 (115–381 mmHg) vs. 132 (95–228) p = 0.016) during the first hour after CA. Corresponding difference persisted during following 23 hours (275, IQR 163–387 vs. 145, IQR 120–221, p < 0.001). Patients with OHCA or ED IHCA had significantly higher P/F-ratios than other CA patients both during the first hour (211, IQR 119–405 vs. 158, 98–235, p = 0.045) and the following 23 hours (367, IQR 206–489 vs. 230, 160–312, p < 0.001). sixty-four patients suffered an OHCA or ED IHCA, 37 patients were exposed to higher PaO_2_ and 23 patients had higher FiO_2_ outside the ICU. In a multiple logistic regression analysis of patients that received mechanical ventilation including the following variables; arrest location, time to return of spontaneous circulation and the location of the arrest, the location was the only independent predictor of hyperoxia exposure (p = 0.006, OR 4.6 (95% CI 1.6-13.8)).

**Table 3:** Description of post-cardiac arrest factors in patients exposed to hyperoxia and those not exposed to hyperoxia

	**All**	**Not exposed to hyperoxia**	**Hyperoxia exposure**	**P-value**
	**n = 119**	**n = 70**	**n = 49**	
**Factors after cardiac arrest**				
Lowest median arterial blood pressure (MAP)	68 (53–80)	71 (55–84)	60 (48–78)	0,133
pH	7,12 (6,98- 7,26)	7,12 (6,98- 7,26)	7,11 (6,98- 7,26)	0,897
Lactate (mmol/l)	8,70 (4,60- 11,80)	7,30 (4,00- 12,00)	9,25 (4,78- 11,78)	0,282
Glucose (mmol/l)	11,65 (8,23- 15,80)	10,45 (7,05- 14,95)	13,80 (9,50- 17,00)	0,017
**Respiratory management and arterial blood gases**				
Mechanical ventilation	96% (114)	93% (65)	100% (49)	0,056
Duration of mechanical ventilation				
Less than 24 hours	31% (37)	31% (20)	35% (17)	0,658
Longer than 24 hours	65% (77)	69% (45)	65% (32)	
**ICU management**				
Delay to ICU admission (hrs)	3 (0,5-5)	1 (0,5- 5)	4 (3–6)	0,003
Therapeutic hypothermia	30% (36)	20% (14)	45% (22)	0,004
Coronary intervention	40% (48)	33% (23)	31% (25)	0,854
**Severity of illness scores**				
Apache III score (ICU admission)	101 (70–118)	103 (70–116)	98 (73,5- 124)	0,638
**Outcome**				
ICU discharge	49% (58)	46% (32)	53% (26)	0,430
ICU length of stay	3,6 (1,4- 6,1)	3,7 (1,9- 6,6)	3,6 (0,8- 6,0)	0,474
Thirty day survival	39% (47)	36% (25)	45% (22)	0,313
Hospital discharge	37% (44)	34% (24)	41% (20)	0,468

### ICU care

Therapeutic hypothermia was used in 36 out of a total of 119 patients, in whom 22 (45%) were exposed to hyperoxia and 14 (20%) were not exposed (Table 3). For measured laboratory parameters after the arrest the only notable difference between those exposed and those not exposed to hyperoxia was the measured glucose level, with hyperoxic patients having a higher mean glucose value (13.8 mmol/l) than non-hyperoxic patients (10.5 mmol/l) (Table 3). The severity of illness is presented as Apache III scores. The average Apache scores were 101, with no difference between those exposed and those not exposed to hyperoxia (Table 3). Length of stay at the ICU was equal between the groups, and 44 (37%) of all patients were discharged from the hospital (Table 3). There were no statistically significant differences in numbers of patients discharged from the hospital and thirty day survival between patients with hyperoxia exposure and no exposure (Table 3).

## Discussion

In this single centre study we found that hyperoxia is a common phenomenon during the first 24 hours after cardiac arrest. The incidence was higher than in the two previous registry trials and a recent trial focusing on pediatric cardiac arrest [7,8,12]. Hyperoxia exposure is more common following OHCA, which may be related to differences in arrest aetiology, difficulties with monitoring, the use of higher fractions of oxygen than needed, and lack of protocols for adjusting inspired oxygen concentration. There were differences between patients exposed to hyperoxia and those not exposed concerning time to ROSC. The reason for this in unclear, but this might influence survival and should be controlled for in studies investigating the relationship between hyperoxia exposure and survival.

There are conflicting results in studies about the prevalence of hyperoxia and its effects [7,8,12,13]. In a multicentre registry study by Kilgannon colleagues of a cohort of 6326 patients who had survived non-traumatic cardiac arrest admitted to the ICU, 18% were exposed to hyperoxia. In a similar study by Bellomo and colleagues, with a much larger cohort (12,108 pts), hyperoxia occurred in only 10.6% of the patients and isolated hypoxia (PaO_2_ < 60 mmHg, regardless of FiO_2_ level) seemed to be as common as hyperoxia. Janz and colleagues investigated hyperoxia in patients treated with mild therapeutic hypothermia: about 30% of the patients had maximum PaO_2_ values over 300 mmHg during the first 24 hours after ROSC [13]. In a multi-centre study by del Castillo and colleagues, who examined hyperoxia in resuscitated paediatric patients, hyperoxia occurred in only 8.5% of the patients after ROSC [12]. They also looked at oxygen values measured 24 h after the CA and found that the prevalence of hyperoxia was 1.7%. The ethology of cardiac arrests is nevertheless different in pediatric patients compared to adults and paediatricians are more aware of the risks of hyperoxia from neonatal experience [14,15]. The conflicting findings of these studies maybe in the methodology, i.e. Kilgannon and colleagues used the first blood gas measured in the ICU whereas Janz and del Castillo and colleagues used the highest oxygen value measured in the ICU during the first 24 hours after the CA. Bellomo and colleagues on the other hand used the worst oxygen value of the first 24 hours. In our study, in which we used the highest arterial oxygen value measured during the first 24 hours after ROSC, 41.2% were exposed to hyperoxia. This is probably related to the fact that we also included blood gas values obtained prior to ICU admission and thus previous trials may have underestimated the true prevalence of hyperoxia exposure in patients treated in the ICU following cardiac arrest. In the study by Kilgannon and colleagues, 63% of the patients where exposed to hypoxia, and in Bellomo and colleagues study as many as 73.5% [7,8]. The rate of hypoxia was only 14% in our study and it differs considerably from the rates in the previous investigations. Hyperoxia may be an unintentional result of strictly avoiding hypoxia and according to the study by Kilgannon and colleagues it is associated with higher mortality than hypoxia [7].

In this trial 60% of OHCA patients were exposed to oxygen values higher than 300 mmHg. This reflects the use of higher oxygen fractions prehospital and in the emergency department. Mechanically ventilated patients in the ED might receive less attention than those in the ICU and monitoring difficulties might such as the use peripheral oxygen saturation may contribute. It is also possible that the initial ventilation perfusion mismatch in OHCA patients is more rapidly corrected after the arrest and thus maintaining high oxygen fractions results in a higher likelihood of hyperoxia exposure. Therefore diligent reassessment of ventilator setting is of high priority. Interestingly in the present trial delay to ICU admission was associated with hyperoxia exposure. This supports the notion of a more diligent follow-up of mechanical ventilation settings in the ICU than in the ED, with titration of inspired oxygen to lower fractions when a sufficient SpO_2_ level is reached. This may be of importance since overcrowding of EDs and ICUs is not uncommon.

According to the current resuscitation guidelines by the American Heart Association, 100% oxygen should be used during initial resuscitation, but after ROSC, the inspired oxygen should be titrated to the lowest level required to achieve an arterial oxygen saturation of ≥94% [10]. The exact SpO_2_ goal in critically ill patients is unknown. Recently Smith and colleagues argued that a SpO_2_ goal of 94% in ward patients might be too low [16]. They suggest that that the lower SpO_2_-range should be reassessed to 96%, because in their study the majority of the patients had SpO_2_-values >96% and many of them were acutely ill, and it did not increase mortality, but the upper target of the range should be 98% until the controversies surrounding hyperoxia are resolved. The use of pulseoximetry immediately following ROSC may be unreliable and give underestimated SpO_2_ values because of decreased peripheral perfusion and possible unstable haemodynamic status. Alternative methods, such as cerebral oximetry or pulseoxymeter with a transcutaneous forehead SpO_2_ sensor might come in question. The use of cerebral oximetry was found to be feasible during IHCA and OHCA [17,18].

We also found that patients with a long delay to ROSC were more likely to suffer of arterial hyperoxia but ROSC delay was not an independent predictor of hyperoxia exposure and thus may be related to the fact that OHCA patients in general have longer ROSC delays. How the prolonged latency to ROSC simultaneously with hyperoxia influences on survival is largely unknown since in the studies by Bellomo and Kilgannon the time to ROSC was not reported. This is paramount and should be taken into account when investigating the association between hyperoxia and mortality.

Induced mild therapeutic hypothermia has been proved to improve neurological outcomes for unconscious adult patients with ROSC after out-of-hospital VF CA [19]. Janz and colleagues have indicated an association between hyperoxia and in-hospital mortality in CA patients undergoing mild therapeutic hypothermia [13]. They found that patients with higher levels of maximum PaO_2_ during the first 24 hours after ROSC had increased in-hospital mortality and a more unfavourable neurologic outcome. In our study therapeutic hypothermia was more often induced in patients suffering from hyperoxia, with 45% treated with hypothermia. How arterial hyperoxia influences outcome in patients treated with therapeutic hypothermia in CA patients needs further studies.

Hyperglycaemia is common after resuscitation from CA arrest and it is generally believed to be the result of a stress response. In our trial we found a statistically significant difference in glucose values between patients exposed and those not exposed to hyperoxia: hyperoxic patients had higher values than non hyperoxic patients. The difference may not be causal and it may be explained by that patients suffering from hyperoxia had an OHCA with prolonged resuscitation resulting in a greater stress response. But further evaluation of this is warranted, as in an animal study, hyperoxia-induced hyperglycemia has been seen in newborn piglets, during ventilator and cardiopulmonary bypass (CPB) and in the absence of CPB [20,21]. With reduction of the oxygen levels to normoxia the hyperglycemic response in the newborns abolished.

## Study limitations

The main limitation of this study is that it is from a single centre and the small study size, even so we feel that the main findings are applicable to other centres as well. In addition we had to divide the patient data into two groups; those exposed to hyperoxia and those not exposed, thus some of those not exposed had oxygen values indicating hypoxia. The data was collected in 2008–2010, when there were no specific guidelines for “the upper limit” of oxygen and may not reflect clinical practise after the introduction of the latest resuscitation guidelines.

## Conclusions

In this single centre study, arterial hyperoxia was a common finding. It usually occurred in out-of-hospital-cardiac-arrest-patients with a prolonged time to ROSC, and a long delay to ICU admission. The impact of hyperoxia requires evaluation and it should be controlled for in cardiac arrest studies. Improvements in the monitoring of oxygenation and education on adjustment of inspired oxygen concentration require future study.

## Competing interests

We declare that there is no financial or other competing interest for any of the authors.

## Authors’ contributions

MBS and MP designed the study. The data was collected by MBS. The data was analysed by AN who drafted the manuscript. MBS and MP revised the manuscript draft. All authors have read and approved the final version.
